# Supplementary material for: Prolonging lung cancer response to EGFR inhibition by targeting the selective advantage of resistant cells
Source: Nat Commun. 2025 Aug 22;16:7853. doi: 10.1038/s41467-025-61788-w (PMC12373916; doi:10.1038/s41467-025-61788-w)
Supplement: Supplementary file 1 — Supplementary Information [file 41467_2025_61788_MOESM1_ESM.pdf]

Supplementary information

Prolonging lung cancer response to EGFR inhibition by targeting the selective advantage of resistant cells

Lisa Brunet[1,2], #David Alexandre[1,2], #Jiyoung Lee[1,2], Maria del Mar Blanquer-Rosselló[1,2], David Bracquemond[3], Alexis Guernet[1,2], Houssein Chhouri[1,2], Mathilde Goupil[1,2], Zoulika Kherrouche[4], Arnaud Arabo[5], Maicol Mancini[3], Dorthe Cartier[1,2], Shen Yao[6], David Godefroy[1,2], Julie Dehedin[1,2], Jian-Rong Li[7,8], Céline Duparc[1,2], Philippe Jamme[4], Audrey Vinchent[4], Caroline Bérard[9], David Tulasne[4], Sabrina Arena[10,11], Alberto Bardelli[10,12], Chao Cheng[7,8], Byoung Chul Cho[13], Olivier Wurtz[14], Cédric Coulouarn[15], Antonio Maraver[3], Stuart A. Aaronson[6], Alexis B. Cortot[4,16], Youssef Anouar[1,2] and Luca Grumolato[1,2,*]

[1]Univ Rouen Normandie, INSERM, NorDiC UMR 1239, Rouen, France.
[2]Institute for Research and Innovation in Biomedicine, Rouen, France.
[3]Institut de Recherche en Cancérologie de Montpellier (IRCM), Inserm, Université de Montpellier, Institut Régional du Cancer de Montpellier (ICM), Montpellier, France.
[4]Univ. Lille, CNRS, Inserm, CHU Lille, Institut Pasteur de Lille, UMR9020 – UMR1277 - Canther – Cancer Heterogeneity, Plasticity and Resistance to Therapies, Lille, France.
[5]Univ Rouen Normandie, INSERM, CNRS, Normandie Université, HeRacLeS US51 UAR2026, SRB, Rouen, France.
[6]Department of Oncological Sciences, Tisch Cancer Institute, Icahn School of Medicine at Mount Sinai, New York, NY, USA.
[7]Department of Medicine, Baylor College of Medicine, Houston, TX 777030, USA.
[8]Institute for Clinical and Translational Research, Baylor College of Medicine, Houston, TX 777030, USA.
[9]Univ Rouen Normandie, LITIS EA 4108, Rouen, France.
[10]Department of Oncology, University of Torino, TO, Italy.
[11]Candiolo Cancer Institute, FPO - IRCCS, 10060 Candiolo, TO, Italy.
[12]IFOM ETS - The AIRC Institute of Molecular Oncology, 20139 Milan, Italy.
[13]Division of Medical Oncology, Yonsei Cancer Center, Yonsei University College of Medicine, Seoul, Republic of Korea.
[14]Univ Rouen Normandie, INSERM, U1245, Cancer and Brain Genomics, Rouen, France.
[15]Inserm, Univ Rennes, UMR_S 1242, Oncogenesis Stress Signaling (OSS) laboratory, Centre de Lutte contre le Cancer Eugène Marquis, Rennes, France
[16]Univ. Lille, CHU Lille, Thoracic Oncology Department, Lille, France.

# These authors contributed equally
* Correspondance: luca.grumolato@univ-rouen.fr

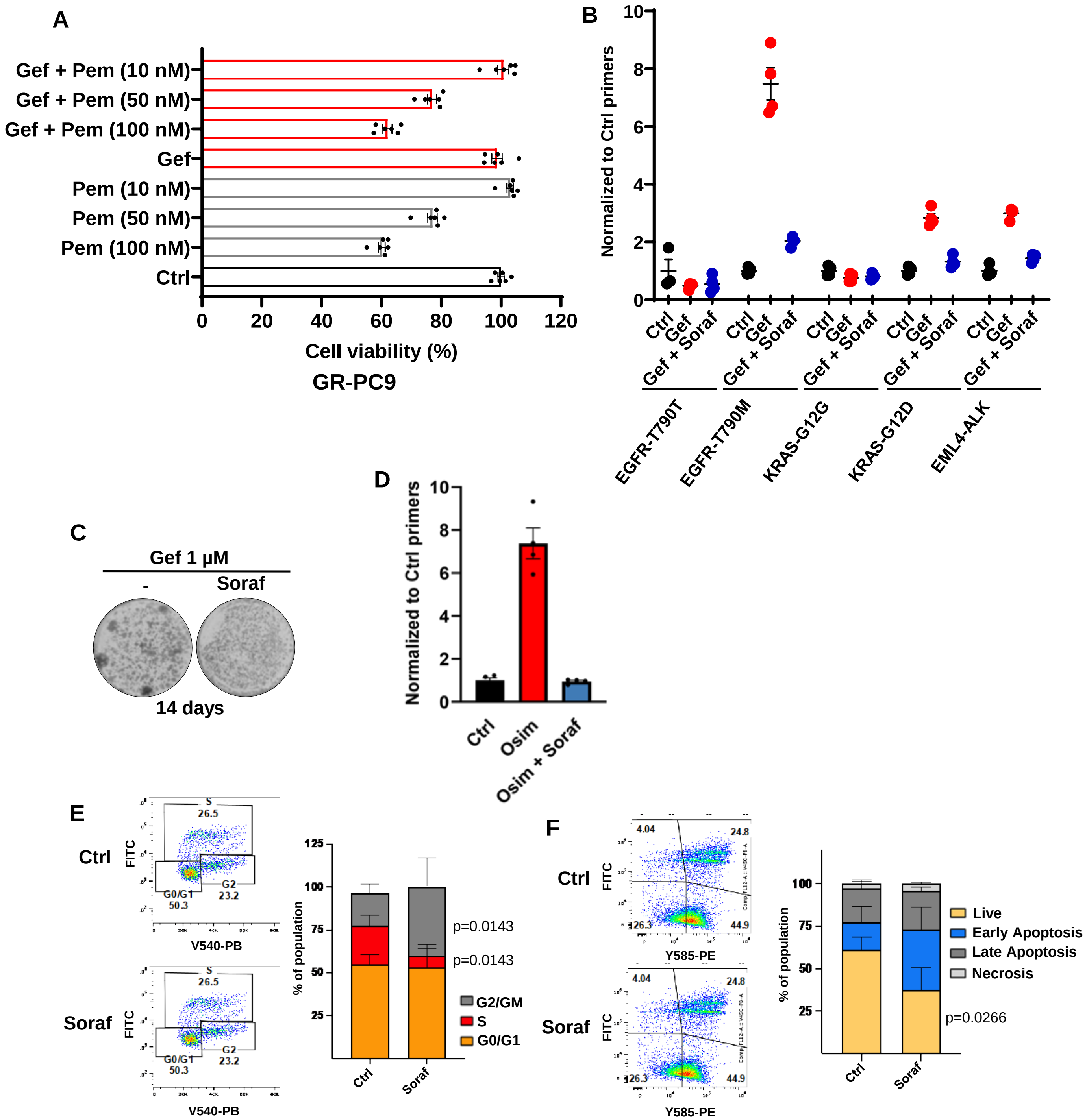

Supplementary Fig.1. Effects of sorafenib in NSCLC cells.

A, Cell viability assay of gefitinib-resistant (EGFR-T790M) PC9 cells treated for 5 days with gefitinib (1 µM) or pemetrexed (100, 50 or 10 nM). The fraction of viable cells was measured by CellTiter-Glo and normalized to the DMSO-treated control. The five biological replicates per condition and their mean ± SEM are shown. The panel illustrates one representative of three independent experiments.

B, The indicated CRISPR-barcodes were introduced in PC9 cells, and the cells were treated with or without gefitinib (1 µM), alone or in combination with sorafenib (5 µM), for 5 days. The proportion of the barcodes was measured by qPCR from genomic DNA and normalized using EGFR_Ctrl primers. The four biological replicates per condition and their mean ± SEM are shown. The panel illustrates one representative of three independent experiments.

C, Representative images from three independent experiments of colony forming assays of the population of CRISPR-barcoded PC9 cells shown in **B** treated with gefitinib (1 µM) alone or in combination with sorafenib (5 µM) for 14 days.

D, CRISPR-barcoding was used to introduce the EGFR-C797S barcode in a population of PC9 cells containing the EGFR-T790M mutation. The cells were then treated with osimertinib (1 µM) alone or with sorafenib (5 µM) for 9 days, and the proportion of the EGFR-C797S barcode was assessed by qPCR. The four biological replicates per condition and their mean ± SEM are shown. The panel illustrates one representative of four independent experiments.

E-F. HCC827 cells were treated with or without sorafenib (5 µM) for 72h and cell cycle was analyzed by flow cytometry by quantifying the incorporation of EdU (**E**) and apoptosis was analyzed by annexin V staining (**F**). One FACS diagram representative of three independent experiments is shown. The histograms represent the mean ± SEM of three independent experiments (Mann-Whitney one-tailed test). Source data are provided as a Source Data file.

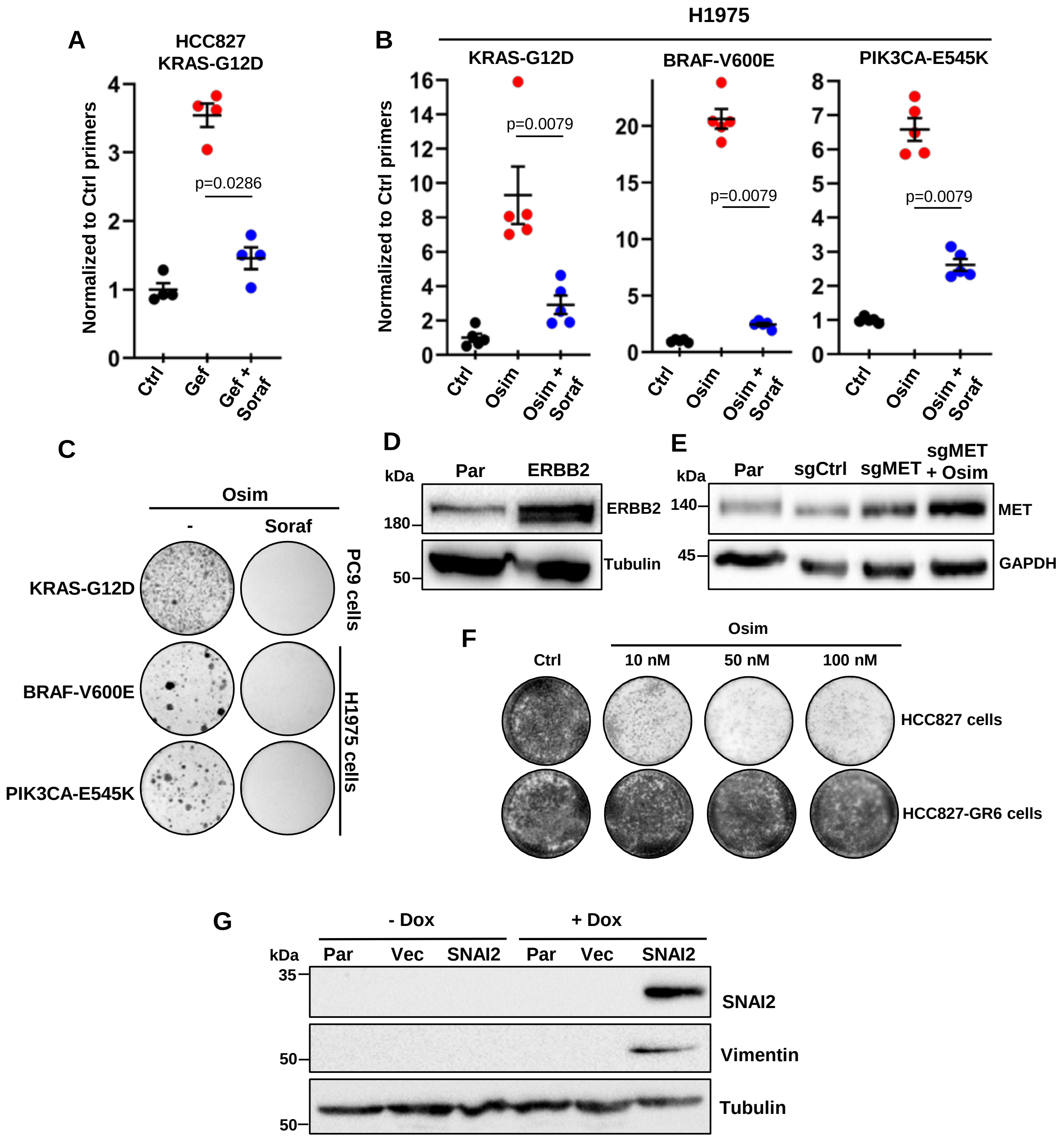

Supplementary Fig.2. **Effects of Sorafenib on different mechanisms of resistance to EGFR-TKIs.**

A, Effects of gefitinib (1 µM), with or without sorafenib (5 µM), on the proportion of HCC827 cells containing the CRISPR-barcode KRAS-G12D after 5 days of treatment. The barcode levels were assessed by qPCR and normalized using EGFR_Ctrl primers. The four biological replicates per condition and their mean ± SEM are shown from one experiment (Mann-Whitney two-tailed test).

B, Effects of osimertinib (0,1 µM) with or without sorafenib (5 µM) on the proportion of KRAS-G12D (15 days), BRAF-V600E (7 days) or PIK3CA-E545K (15 days) CRISPR-barcodes in H1975 cells. The four biological replicates per condition and their mean ± SEM are shown. The panel illustrates one representative of three independent experiments (Mann-Whitney two-tailed test).

C, PC9 and H1975 cells containing the indicated CRISPR-barcodes were treated with osimertinib alone (0,1 µM) or in combination with sorafenib (5 µM) for one month. The cells were then fixed and stained with crystal violet. The panel illustrates one of three biological replicates per condition and it's representative of two (H1975) or three (PC9) independent experiments.

D, PC9 cells were transduced (ERBB2) or not (Par) with a ERBB2 lentivirus and immunoblot was performed using the indicated antibodies. Representative blots from n = 2 independent experiments.

E, The levels of MET receptor were assessed by immunoblot in parental (Par) PC9 and in cells containing the dCas9 activator system with a control (sgCtrl) or a MET-specific (sgMET) sgRNA. A population of sgMET cells was selected for two weeks with osimertinib (0,1 µM; sgMET+Osim) before the lysis (n=1).

F, HCC827 and HCC827-GR6 cells were treated for 7 or 5 days, respectively, with the indicated concentrations of osimertinib, followed by fixation and crystal violet staining (n=3 biological replicates).

G. PC9 cells were transduced with an empty lentiviral vector (Vec) or a vector for inducible expression of the EMT transcription factor SNAI2 (SNAI2), followed by a 7 day treatment in the presence or the absence of doxycycline (1 µg/ml), followed by immunoblot using indicated antibodies. Parental PC9 (Par) were used as negative control. Representative blots from n = 2 independent experiments. Source data are provided as a Source Data file.

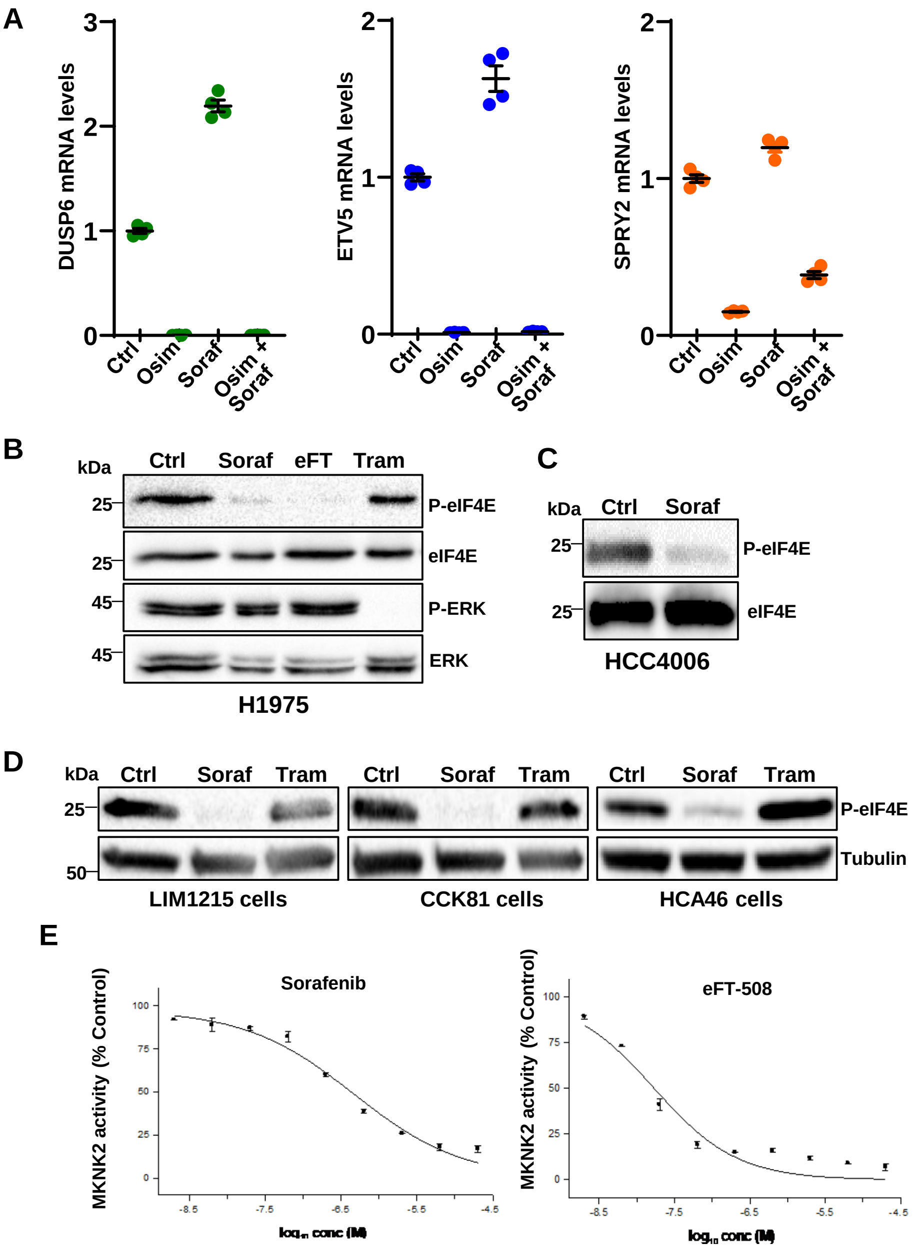

F

Assay	Compound	IC50
MKNK1	Sorafenib	6.0E-06 M
	eFT-508	2.4E-09 M
MKNK2	Sorafenib	4.62E-07M
	eFT-508	1.7E-08M

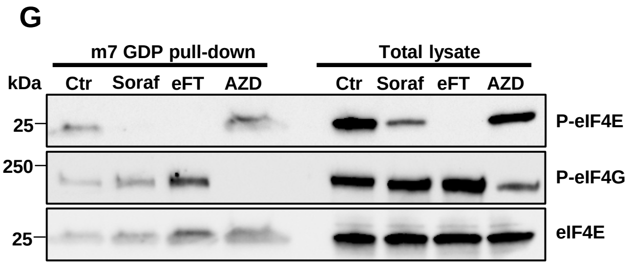

Supplementary Fig.3. Sorafenib blocks phosphorylation of eIF4E by a direct inhibition of MKNK activity without affecting MAPKs in NSCLC cells.

A, PC9 cells were treated with osimertinib (1 µM) and sorafenib (5 µM), alone or in combination, for 2 days and the expression of DUSP6, SPRY2 and ETV5 was assessed by qPCR. The four biological replicates per condition and their mean ±SEM are shown. The panel illustrates one representative of two or three independent experiments.

B, H1975 and HCC4006 NSCLC cells were treated for 2h with or without sorafenib (5 µM), eFT-508 (1 µM; eFT, MKNK inhibitor) or trametinib (50 nM; Tram, MEK inhibitor), followed by immunoblot using the indicated antibodies. Representative blots from n=3 independent experiments.

C, HCC4006 NSCLC cells were treated for 2h with or without sorafenib (5 µM), followed by immunoblot using the indicated antibodies (n=1).

D, LIM1215, HCA46 and CCK81 CRC cells were treated for 2h in the presence or the absence of sorafenib (3 µM) or trametinib (25 nM), followed by immunoblot using anti-phospho-eIF4E or anti-tubulin antibodies (n = 1 for HCA46 and CCK81 cells and n = 2 for LIM1215 cells).

E, Effects of sorafenib and eFT-508 on the *in vitro* catalytic activity of MKNK2, measured using as a substrate the myelin basic protein.

F, Table reporting the *in vitro* IC50 of sorafenib and eFT-508 for MKNK1 and MKNK2, measured using as a substrate the myelin basic protein or a peptide derived from the human cAMP Response Element Binding protein.

G, Cap pull-down assay in PC9 cells treated for 6h in the presence or the absence of sorafenib (5 µM), eFT-508 (1 µM) or AZD8055 (1 µM; AZD, mTOR inhibitor). Cell lysate was pulled-down using m7 GDP beads, and immunoblot was performed using the indicated antibodies. Representative blots from n = 3 independent experiments. Source data are provided as a Source Data file.

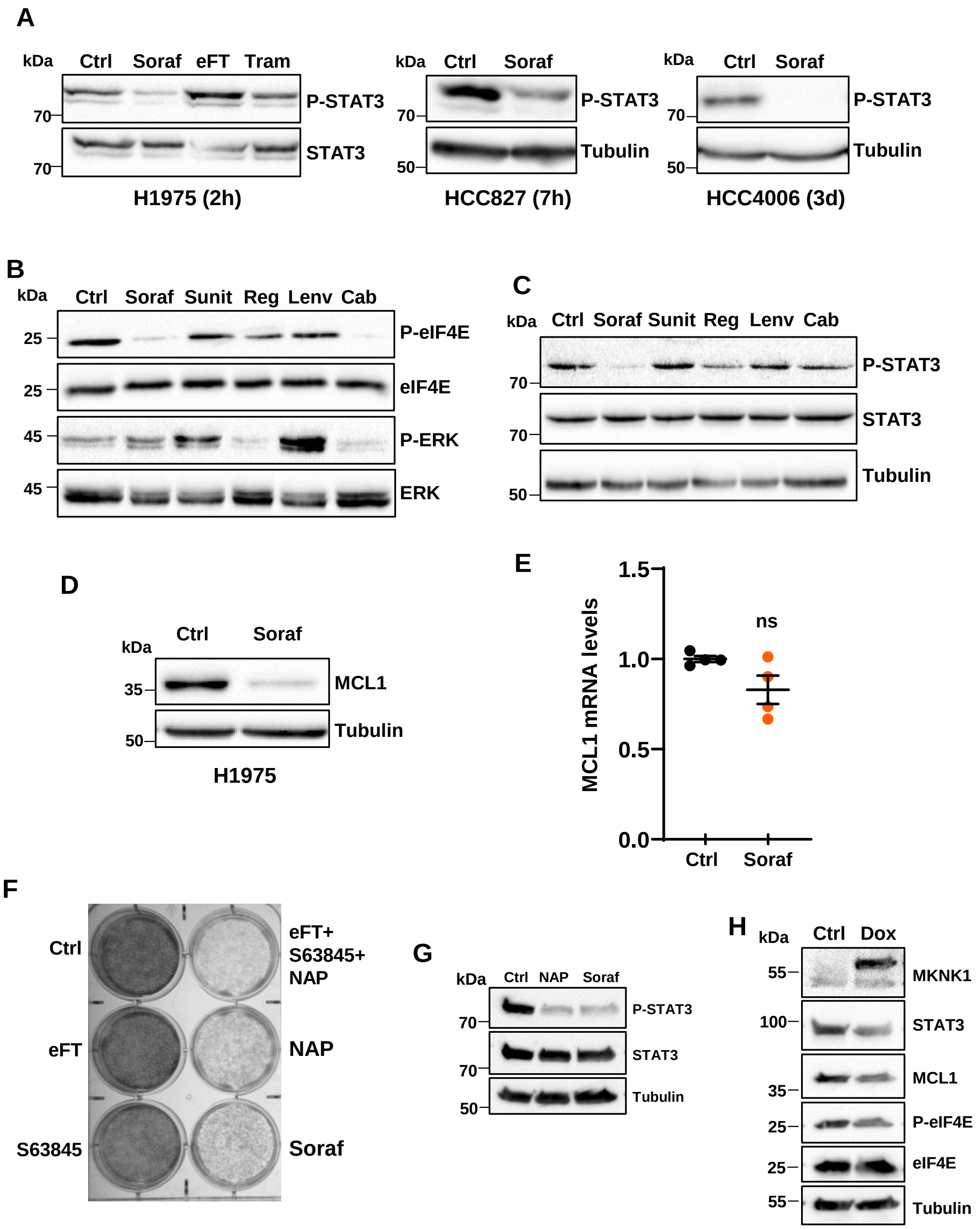

Supplementary Fig.4. Sorafenib inhibits phosphorylation of STAT3 and expression of MCL1 in NSCLC cells.

A, H1975, HCC827 and HCC4006 cells were treated for the indicated time points with sorafenib (5 µM), eFT-508 (1 µM) or trametinib (50 nM), followed by immunoblot using anti-phospho-STAT3, anti-STAT3 or anti-tubulin antibodies (n=1 for H1975 cells and n=2 indepented experiments for HCC827 and HCC4006).

B-C, PC9 cells were treated for 2h (**B**) or 6h (**C**) with sorafenib (5 µM), sunitinib (1 µM), regorafenib (2 µM), lenvatinib (1 µM) or cabozantinib (5 µM) and immunoblot was performed using the indicated antibodies. Representative blots of two independent experiments.

D, H1975 cells were treated for 3 days in the presence or the absence of sorafenib (5 µM), followed by immunoblot using the indicated antibodies (n=1).

E, PC9 cells were treated for 48h with sorafenib (5 µM) and the mRNA levels of MCL1 were assessed by RT-qPCR. The four biological replicates per condition and their mean ± SEM are shown. The panel illustrates one representative of three independent experiments. (ns: not significative, Mann-Whitney two tailed test).

F, Representative images of colony forming assays of PC9 cells treated for 7 days with sorafenib (5 µM), eFT-508 (1 µM), S63845 (0,1 µM; MCL1 inhibitor), napabucasin (0,5 µM; NAP, STAT3 inhibitor) or a combination of eFT-508, S63845 and napabucasin. (n=4 biological replicates, representative of 3 independent experiments).

G, PC9 cells were treated for two days with sorafenib (5 µM) or napabucasin (0,5 µM; NAP), followed by immunoblot using the indicated antibodies. Representative blots of two independent experiments.

H, Osimertinib resistant cells (EGFR-C797S) were transduced with inducible lentiviral vectors containing shSTAT3, shMCL1 and DN-MKNK1 fused to GFP and selected with the three corresponding antibiotics. The cells were then treated for four days with or without doxycycline (2 µg/ml), followed by immunoblot with the indicated antibodies. Source data are provided as a Source Data file.

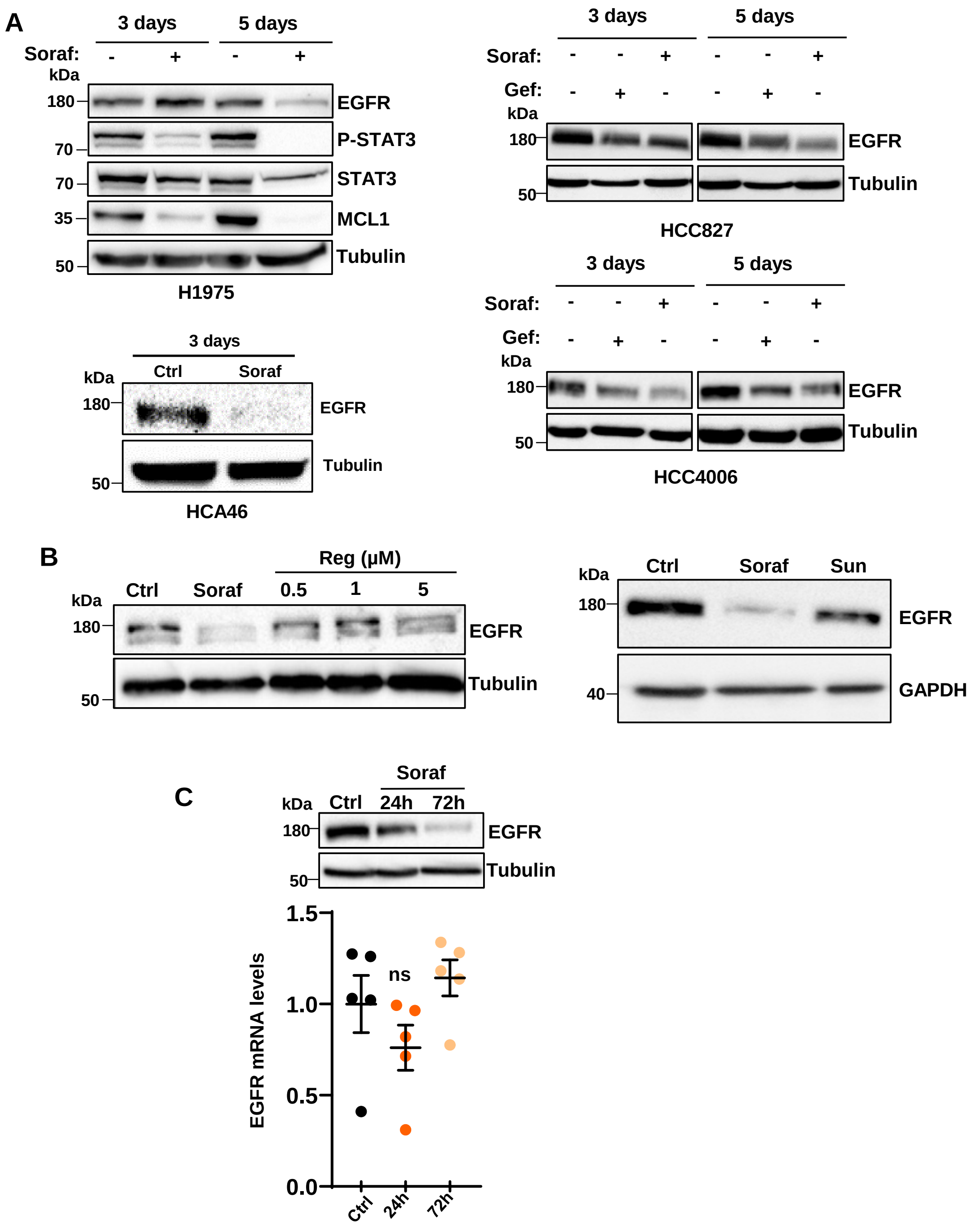

Supplementary Fig.5. **EGFR down-regulation by Sorafenib in NSCLC cells.**

A, H1975, HCC827 and HCC4006 NSCLC cells were treated for 3 and 5 days with sorafenib (5 µM) or gefinitib (1 µM) as indicated, followed by immunoblot using the indicated antibodies. HCA46 CRC cells were treated with sorafenib (5 µM) for 3 days, followed by immunoblot using anti-EGFR or anti-tubulin antibodies (n=1 experiment for H1975 cells and n=2 independent experiments for HCC827, HCC4006 and HCA46 cells).

B, Left panel, PC9 cells were treated for 4 days with sorafenib (5 µM) or the indicated concentrations of regorafenib. Right panel, PC9 cells were treated for 3 days with sorafenib (5 µM) or sunitinib (1 µM). Immunoblot was performed using anti-EGFR, anti-tubulin or anti-GAPDH antibodies (n=1).

C, PC9 cells were treated for 24 and 72h with sorafenib (5 µM) and the levels of EGFR mRNA and protein were assessed by immunoblot (upper panel) or RT-qPCR (lower panel). The four biological replicates per condition and their mean ±SEM are shown. The panel illustrates one representative of three independent experiments. (ns: not significant, $p > 0.05$ Mann-Whitney two tailed test). Source data are provided as a Source Data file.

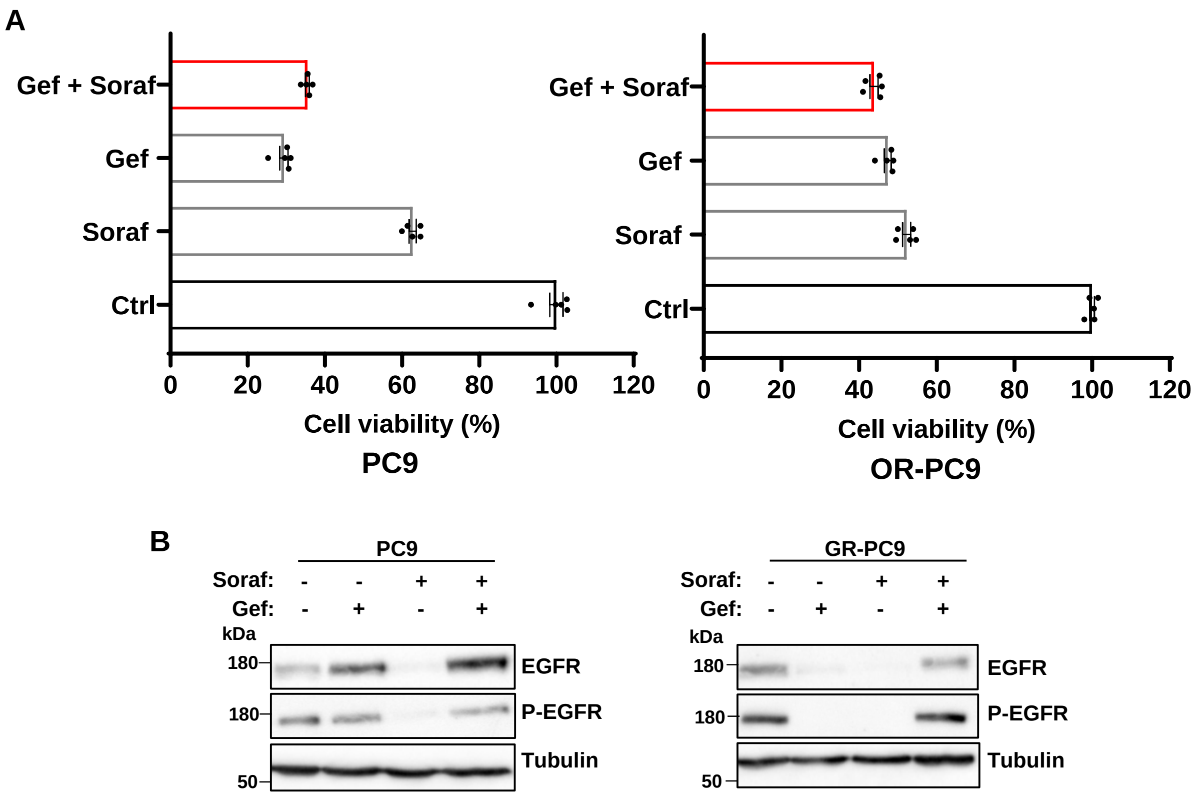

Supplementary Fig.6. Effects of sorafenib on sensitive and EGFR-TKI resistant NSCLC cells.

A, Cell viability assay of parental or osimertinib-resistant PC9 (OR-PC9) cells grown for 5 days in the presence or the absence of gefitinib (1 µM), alone or in combination with sorafenib (5 µM). The fraction of viable cells was measured by CellTiter-Glo and normalized to the DMSO-treated control. The five biological replicates per condition and their mean ±SEM are shown. The panel illustrates one representative of three independent experiments.

B, PC9 and gefitinib-resistant PC9 (GR-PC9) were treated with or without sorafenib (5 µM) in the presence of the absence of gefitinib (1 µM), followed by immunoblot with the indicated antibodies. Representative blots from n = 3 independent experiment. Source data are provided as a Source Data file.

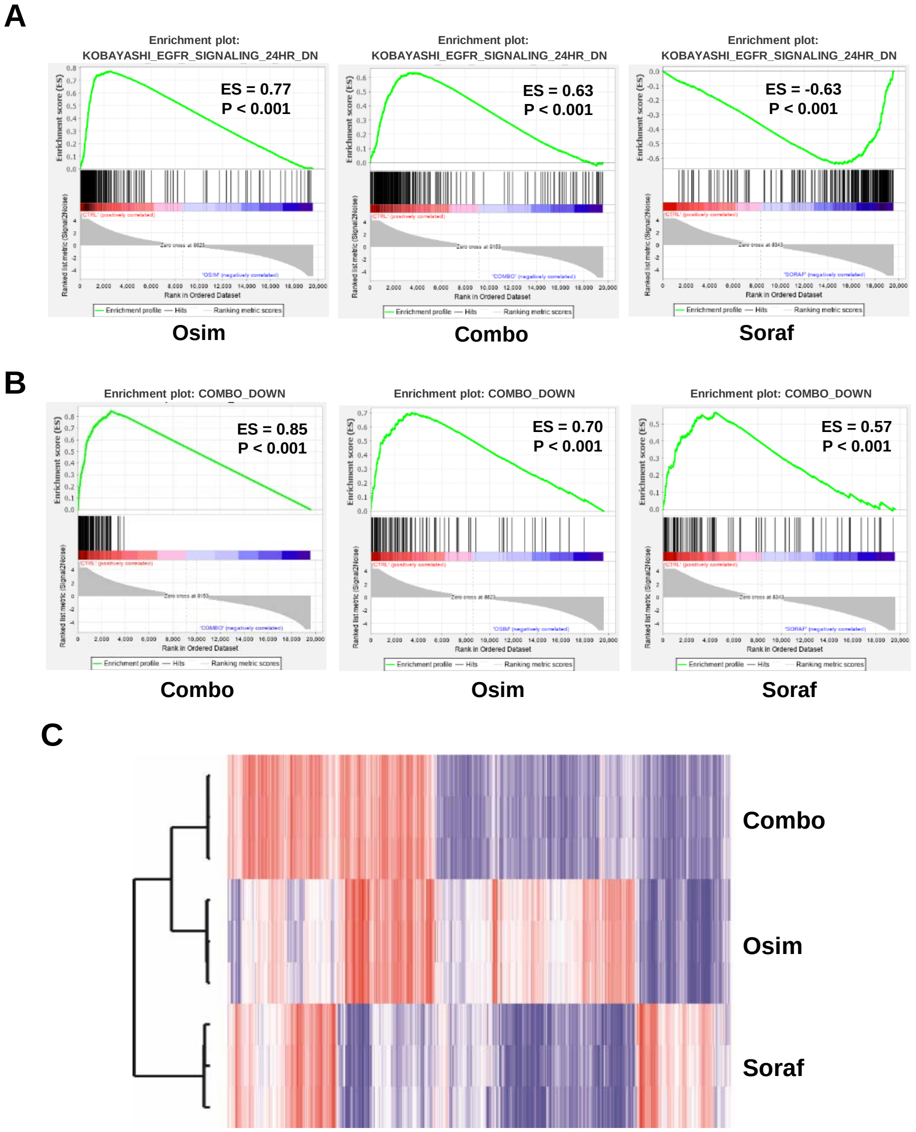

Supplementary Fig.7. Effects of sorafenib on the clonal evolution of NSCLC cells induced by osimertinib.

A, Gene set enrichment analysis (GSEA) of EGFR-TKI down-regulated genes in NSCLC cells (KOBAYASHI_EGFR_SIGNALING_24HR_DOWN), performed on gene array data obtained from PC9 cells treated with osimertinib (1 µM) or sorafenib (5 µM), alone or in combination (Combo), for two days. Enrichment scores (ES) and *p* values are reported.

B, The data described in **A** were analyzed using our osimertinib-sorafenib combination signature (COMBO_DOWN).

C, Heatmap and hierarchical clustering of the gene array data. Source data are provided as a Source Data file.

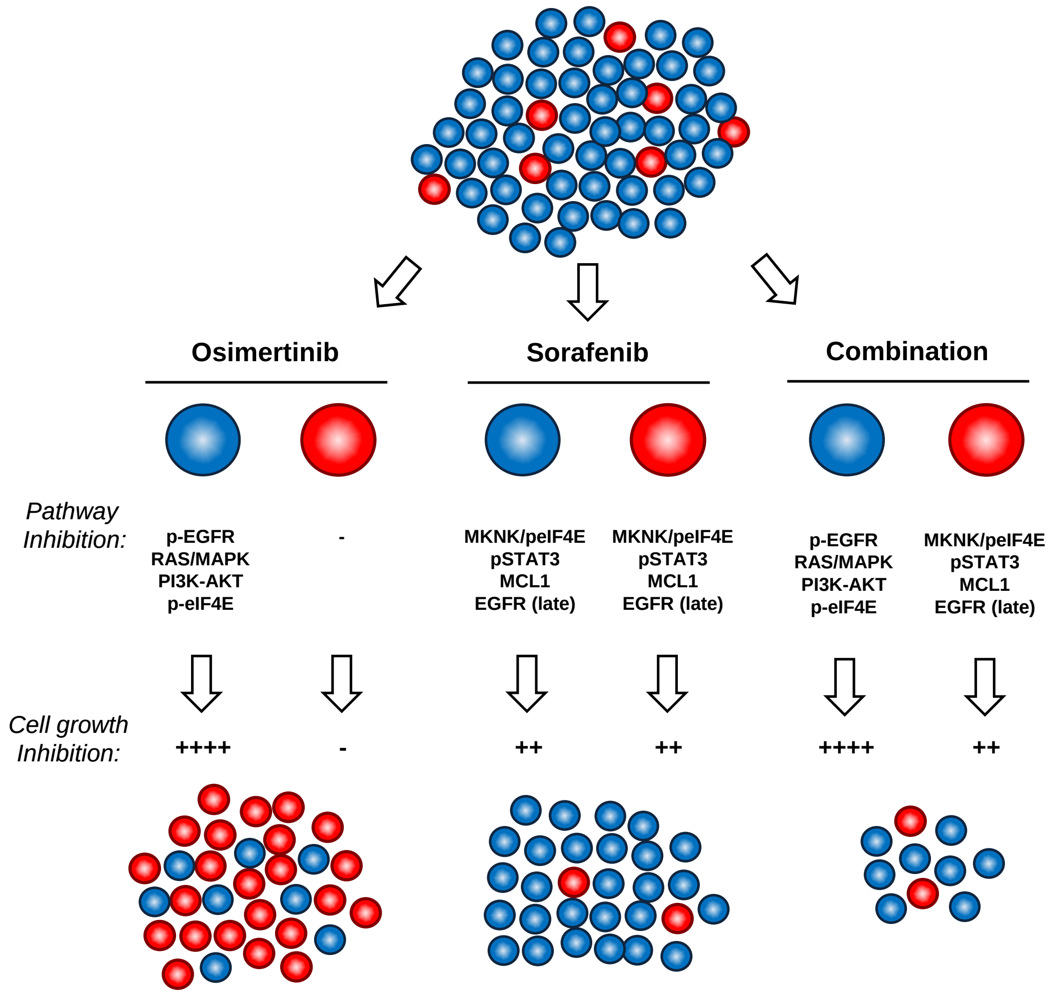

Supplementary Fig.8. Diagram illustrating the effects of osimertinib and sorafenib, alone or in combination, on a mass population of osimertinib-sensitive (blue) and osimertinib-resistant (red) NSCLC cells.

A

	Tumor total volume	Volume of resistant cells	% resistant cells/total volume	fold change vs control
Exp.1 Ctrl	2,34E+11	1,18E+08	0,05	1,00
Exp.1 Osim	3,58E+11	1,14E+09	0,32	6,31
Exp.1 Combo	7,35E+10	8,87E+07	0,12	2,40
Exp.2 Ctrl	8,85E+10	3,83E+08	0,43	1,00
Exp.2 Osim	2,53E+11	2,23E+09	0,88	2,04
Exp.2 Combo	1,05E+11	3,88E+08	0,37	0,85

B

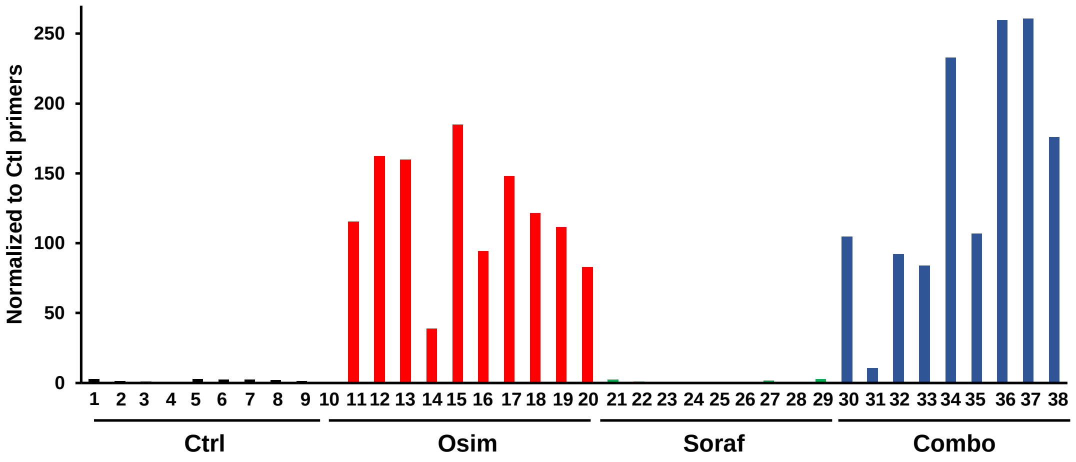

C

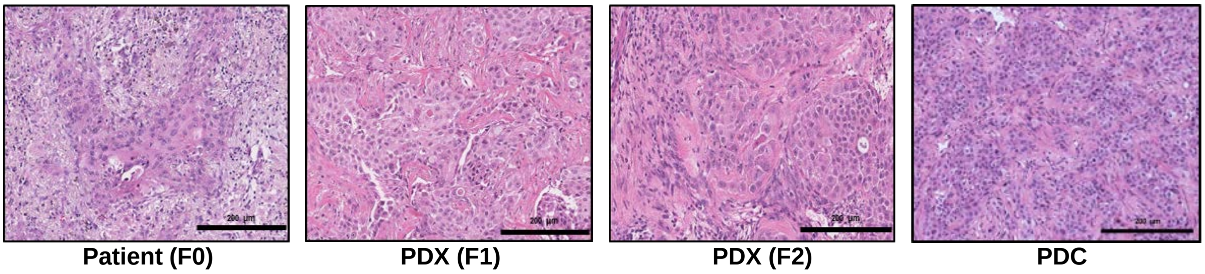

D

	Tumor total volume	Volume of resistant cells	% resistant cells/total volume	fold change vs control
Exp.1 Ctrl	2,96E+11	8,50E+07	0,0287	1,00
Exp.1 Osim	1,85E+11	3,97E+08	0,2145	7,47
Exp.1 Combo	2,03E+11	1,50E+08	0,0740	2,58
Exp.2 Ctrl	2,44E+11	1,21E+07	0,0050	1,00
Exp.2 Osim	3,09E+11	2,93E+09	0,9497	190,88
Exp.2 Combo	1,40E+11	1,55E+07	0,0111	2,23

Supplementary Fig.9. Effects of sorafenib on the emergence of osimertinib resistance *in vivo*.
A, Quantification of the iDISCO experiment shown in Fig. 6A.
B, The levels of the EGFR-C797S CRISPR-barcode were measured by qPCR from genomic DNA derived from the tumors described in Fig. 6B. The values were normalized using control primers (the value of 1 corresponds to the levels measured in the injected cells).
C, Representative images of hematoxylin-eosin staining of the YHIM-1024 original tumor, the corresponding YHIM-1024 PDXs (F1 inoculated in nude mice, F2 in NOG mice) and tumors obtained in SCID mice from YUX-1024 PDCs. Scale bar, 200 µm.
D, Quantification of the iDISCO experiment shown in Fig. 6C. Source data are provided as a Source Data file.

A

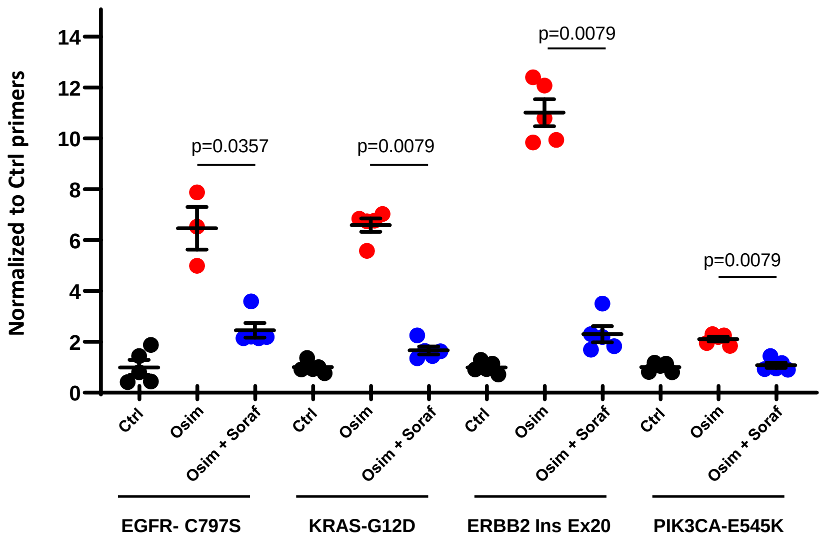

B

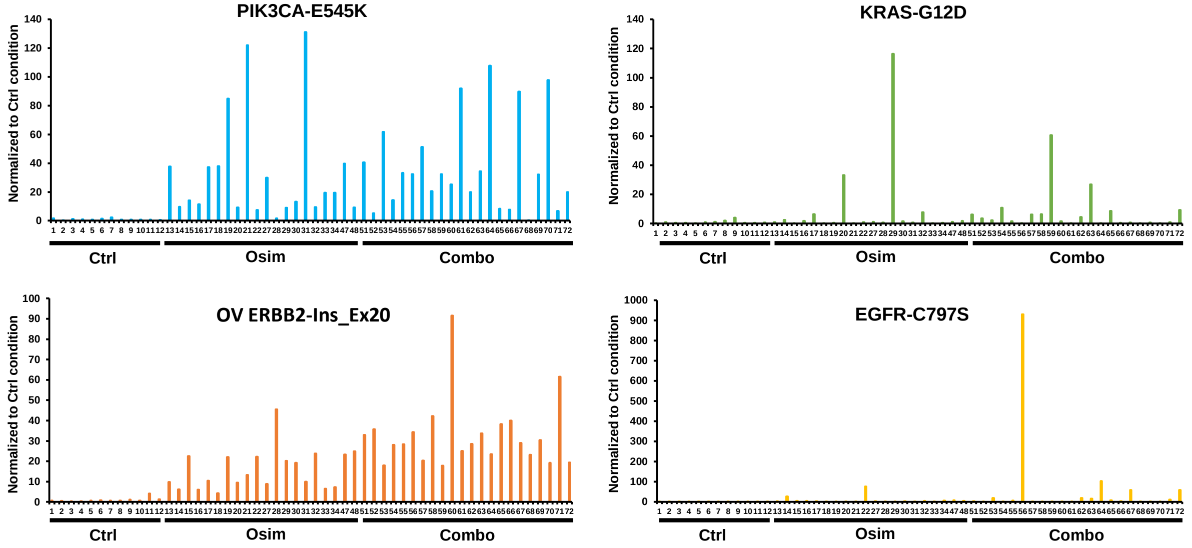

Supplementary Fig.10. Effect of sorafenib on the emergence of osimertinib-resistant subpopulations *in vitro* and *in vivo*.

A, Effects of a 15-day treatment with osimertinib (0,1 µM) with or without sorafenib (5 µM) on the proportion of the indicated barcodes in the same multiplex PC9 cell model used for the mouse experiment described in Fig. 7A,B. The mean ± SEM (n=5 biological replicates) of one representative of three experiments is represented (Mann-Whitney two-tailed test).

B, The levels of the indicated barcodes were measured by qPCR from the tumors described in Fig. 7A-B. Source data are provided as a Source Data file.

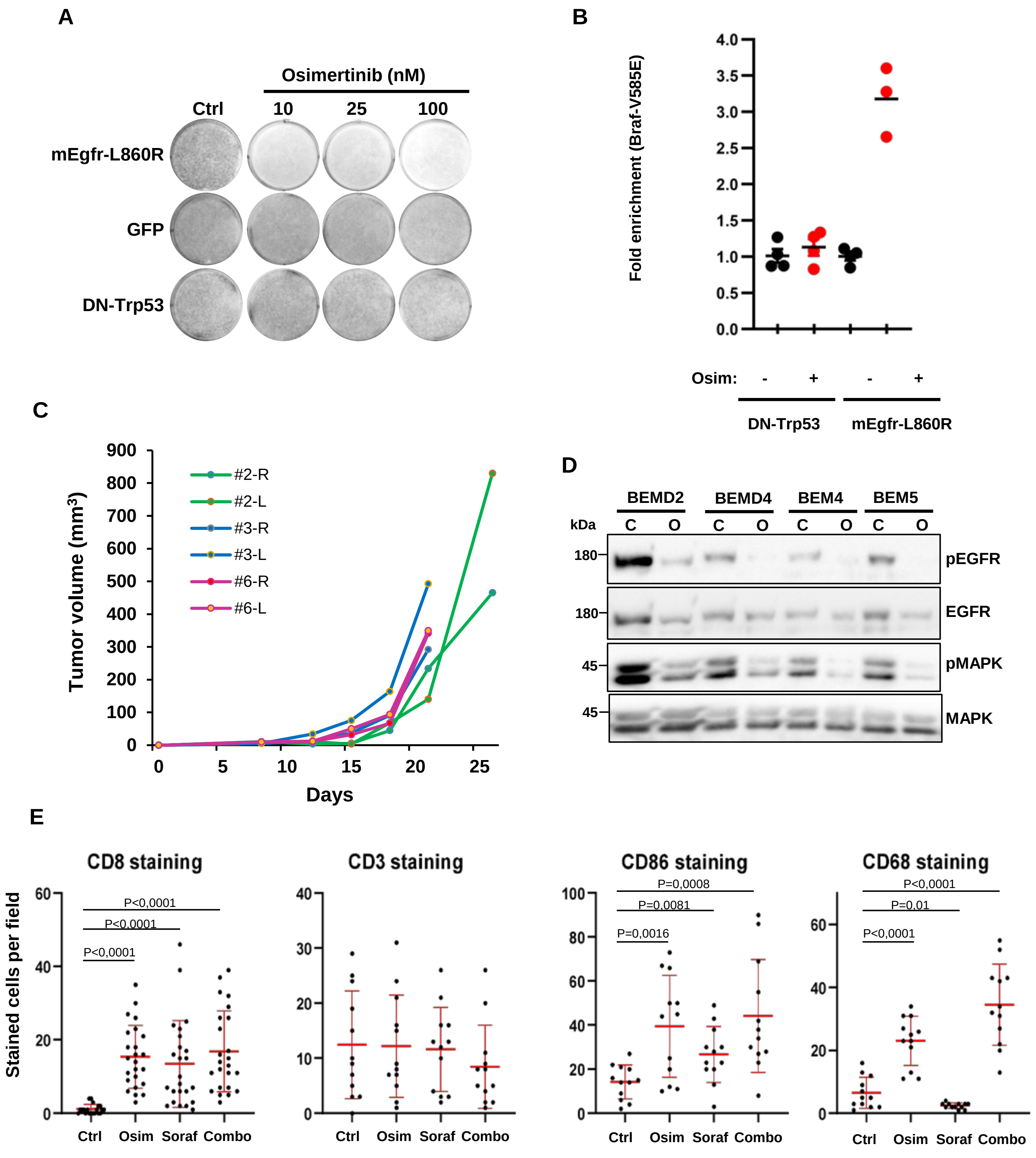

Supplementary Fig.11. Development of a new syngeneic model of oncogenic addiction to mutant EGFR.

A, BALB-3T3 cells expressing mouse Egfr-L860R, GFP or DN-Trp53 were treated for 9 days in 1% calf serum with the indicated concentrations of osimertinib, then fixed and stained with crystal violet (n=1).

B, A small subpopulation of Braf-V585E (corresponding to human BRAF-V600E) cells was generated by CRISPR-barcoding in BALB-3T3-Egfr-L860R or DN-Trp53. The cells were grown for one week in the presence or the absence of osimertinib (100 nM) and the proportion of the Braf-V585E barcode was measured by qPCR from genomic DNA. The three or four biological replicates per condition and their mean ±SEM are shown. The panel illustrates one representative of two independent experiments.

C, BALB-3T3-Egfr-L860R cells were subcutaneously injected in the right (R) and left (L) flanks of three BALB/c mice and the volume of the tumors was measured by caliper.

D, One of the BALB-3T3-Egfr-L860R tumors shown in C was dissected and the cells grown in culture. Different individual clones were isolated (here 4 clones are shown) and treated for two hours in the presence (O) or the absence (C) of osimertinib 100 nM (1% calf serum), followed by immunoblot with the indicated antibodies (n=1).

E, BALB/c mice bearing BEM-5 tumors were treated with osimertinib (20 mg/kg) and sorafenib (60 mg/kg), alone or in combination for ten days, followed by IHC using the indicated antibodies. The number of stained cells per field are indicated (three mice per condition, at least four randomly selected fields per mouse; the *p* values are indicated (t test). Source data are provided as a Source Data file.

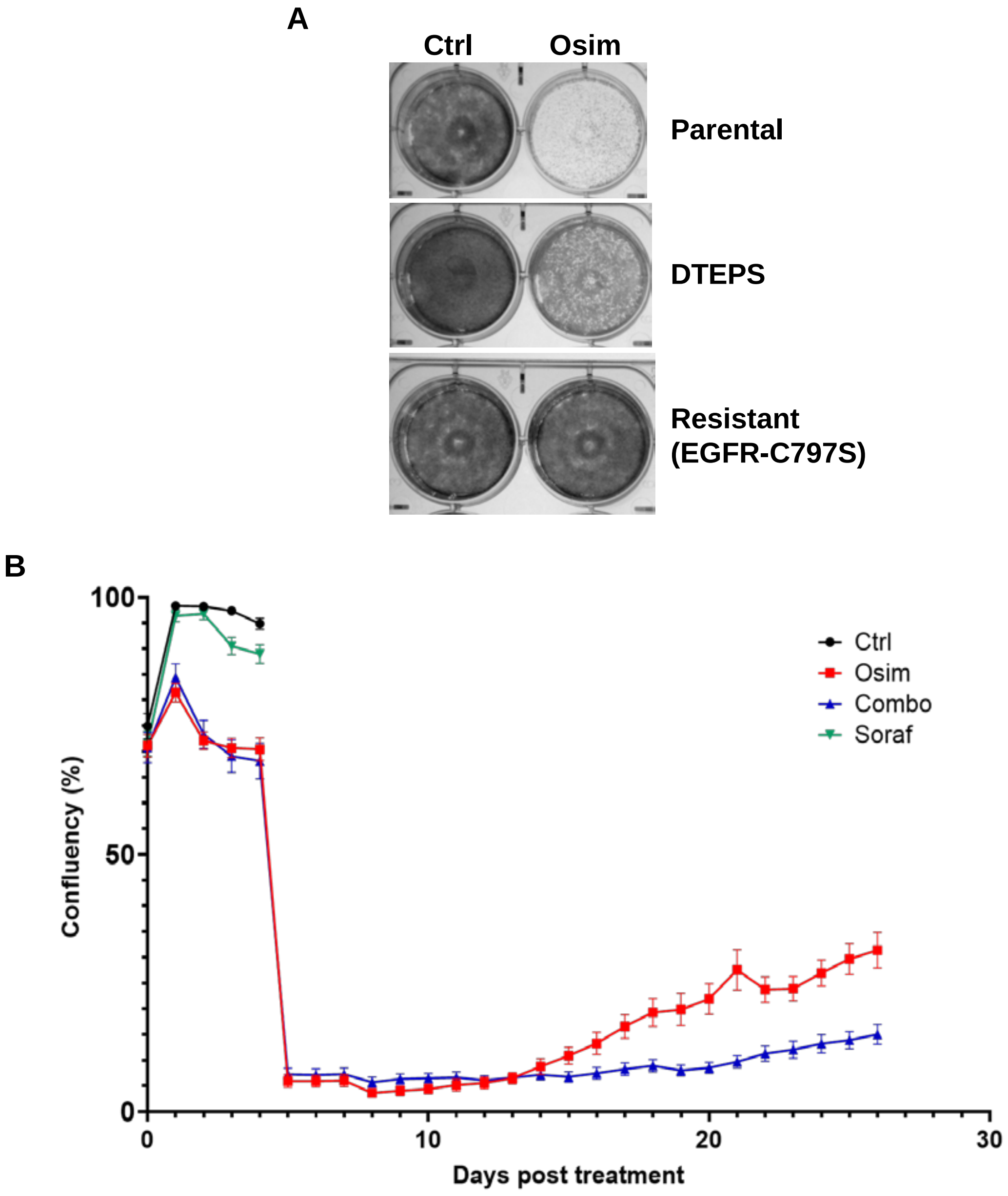

Supplementary Fig.12. Effects of treatment on tolerant/persister cells.

A. Parental, DTEPS or resistant (EGFR-C797S) PC9 cells were treated for 6 days with or without osimertinib (0,1 µM), followed by fixation and crystal violet staining. The panel illustrated one of three biological replicates per condition. The images are representative of two independent experiments.

B. PC9 cells stably expression GFP were treated with osimertinib (0,1 µM) or sorafenib (5 µM), alone or in combination for 26 days. At day 4 after the beginning of the treatment, the control and sorafenib wells reached maximum confluency and all the cells detached. This panel shows the measurements of cell confluency from the experiment shown in Fig.8C. For each time point, the mean ± SEM of seven biological replicates per condition is shown. The panel illustrates one representative of two independent experiments. Source data are provided as a Source Data file.

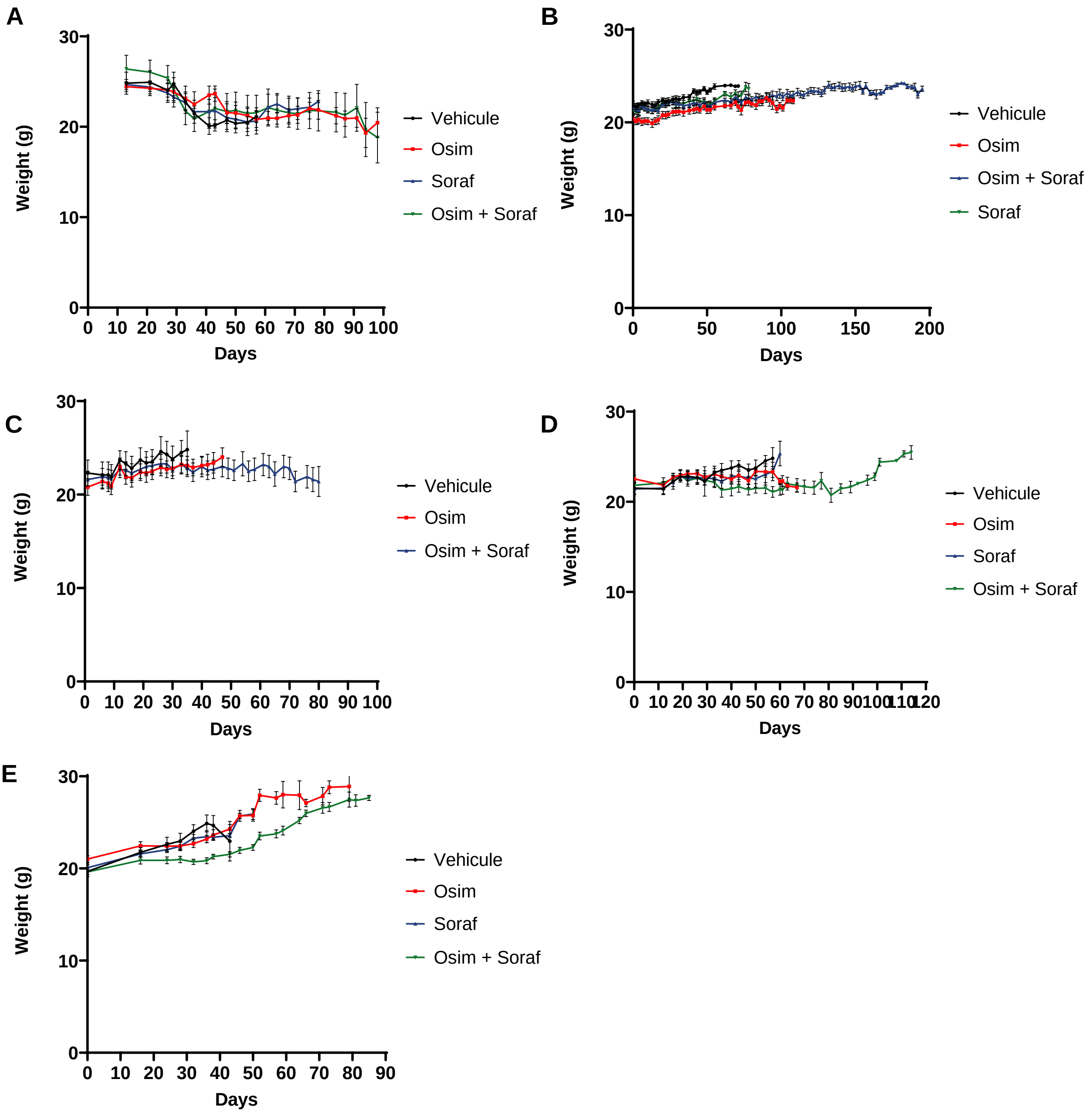

Supplementary Fig.13. Effects of treatment on the body weight of the mice in the different experiments.
A. SCID mice injected with PC9 cells containing a subpopulation of EGFR-C797S cells (Fig. 6B).
B. SCID mice injected with YUX-1024 cells containing a subpopulation of BRAF-V600E cells (Fig. 6D).
C. SCID mice injected with a mass population of PC9 cells containing small pools of EGFR-C797S, KRAS-G12D and PIK3CA-E545K CRISPR-barcoded cells or ERBB2-ex20 overexpressing cells (Fig. 7A).
D. BALB/c mice injected with BEM-5 cells containing a subpopulation of Egfr-C799S cells (Fig. 7E).
E. BALB/c mice injected with BEM-4 cells (Fig. 8F). Source data are provided as a Source Data file.

Supplementary Table 1. List of sgRNA target sequences used for CRISPR-barcoding

Name	Sequence 5’-3’
sgEGFR_T790	CTGCGTGATGAGCTGCACGG
sgEGFR_C797	CATGCCCTTCGGCTGCCTCC
sgEGFR_724	AAGATCAAAGTGCTGGGCTC
sgEGFR_G465	TCCCTCAAGGAGATAAGTGA
sgKRAS	GAATATAAACTTGTGGTAGT
sgEML4	GACCTGAACAGCAAGTTTGT
sgALK	GGCCTTGCTGAAACTTCCTT
sgBRAF	TAGCTACAGTGAAATCTCGA
sgPIK3CA	TCTCTCTGAAATCACTGAGC
sgHER2	AGCATACGTGATGGCTGGTG
sgBRAF (mouse)	ACTCCACCGAGATTTCACTG
sgEGFR_C797 (mouse)	CATGCCCTACGGTTGCCTCC

Supplementary Table 2. List of ssODNs used for CRISPR-barcoding. Mutations compared to the endogenous sequence are indicated in lowercase letters.

Name	Séquence 5'-3'
EGFR-T790M	CTCCCTCCCTCCAGGAAGCCTACGTGATGGCCAGCGTGGACAA<br>CCCCCACGTGTGCCGCCTGCTGGGCATCTGCCTCACCTCtACtGT<br>aCAGCTtATaAtGCAaCTgATGCCCTTCGGCTGCCTCCTGGACTAT<br>GTCCGGGAACACAAAGACAATATTGGCTCCCAGTACCTGCTCAA<br>CTGGTGTGTG
EGFR-C797S	GATGGCCAGCGTGGACAACCCCCACGTGTGCCGCCTGCTGGGC<br>ATCTGCCTCACCTCCACCGTGCAGCTCATCACGCAGCTCATGCC<br>CTTCGGgaGtCTgCTtGAtTAcGTCCGGGAACACAAAGACAATATTG<br>GCTCCCAGTACCTGCTCAACTGGTGTGTGCAGATCGCAAAGGTA<br>ATCAGGGAAG
EGFR-T790M-C797S	GATGGCCAGCGTGGACAACCCCCACGTGTGCCGCCTGCTGGGC<br>ATCTGCCTCACCTCCACCGTGCAGCTCATCAtGCAGCTCATGCCC<br>TTCGGgaGtCTgCTtGAtTAcGTCCGGGAACACAAAGACAATATTGG<br>CTCCCAGTACCTGCTCAACTGGTGTGTGCAGATCGCAAAGGTAA<br>TCAGGGAAG
EGFR-G724S	CCTCTTACACCCAGTGGAGAAGCTCCCAACCAAGCTCTCTTGAG<br>GATCTTGAAGGAAACTGAATTCAAAAAGATCAAgGTcCTtaGtTCCG<br>GTGCGTTCGGCACGGTGTATAAGGTAAGGTCCCTGGCACAGGC<br>CTCTGGGCTGGGCCGCAGGGCCTCTCATGG
EGFR-G465R	TTTCTTCTCTCCAATGTAGTGGTCAGTTTTCTCTTGCAGTCGTCA<br>GCCTGAACATAACATCCTTGGGATTACGCTCCCTCAAGGAaATtA<br>GcGATGGtGAcGTcATtATcTCAaGAAACAAAAATTTGTGCTATGCA<br>AATACAATAAACTGGAAgAAACTGTTTGGGACCTCCGGTCAGAAA<br>ACCAAAATTA
KRAS-G12D	TAACCTTATGTGTGACATGTTCTAATATAGTCACATTTTCATTATTT<br>TTATTATAAGGCCTGCTGAAAATGACTGAATATAAgtTaGTtGTcGT<br>TGGtGCcGacGGCGTAGGCAAGAGTGCCTTGACGATACAGCTAAT<br>TCAGAATCATTTTGTGGACGAATATGATCCAACAATAGAGGTAAA
BRAF-V600E	CTGTTTTCCTTTACTTACTACACCTCAGATATATTTCTTCATGAAG<br>ACCTCACAGTAAAAATAGGTGATTTTGGTCTAGCgACgGaaAAgTC<br>aCGcTGGAGTGGGTCCCATCAGTTTGAACAGTTGTCTGGATCCAT<br>TTTGTGGATGGTAAGAATTGAGGCTATTTT
PI3KCA-E545K	GATATTATTTTATTTTACAGAGTAACAGACTAGCTAGAGACAATGA<br>ATTAAGGGAAAATGACAAAGAACAGCTCAAAGCAATTTCTACACG<br>AGATCCTCTgTCgGAgATaACcaAGCAaGAGAAAGATTTTCTATGG<br>AGTCACAGGTAAGTGCTAAAATGGAGATTCTCTGTTTCTTT
EML4-ALK	AGTGAACACAGTTGTGTTGTTCAATTTTTAAGGTATTTTTAGATGA<br>TAAATATTGATGTAAGTGGAGACAGTTGACCTGAACAGCAAGTTC<br>TTAGGCTCCATGGCACCCAGGGTGCTTCCACCCAACCTTCCCTC

	CCTCCCTCGTTCACGTGGGGTTATACTTGCAACACAGTCTGCTGG
HER2_Ins_Ex20	GGGTGTGTGGTCTCCCATACCCTCTCAGCGTACCCTTGTCCCCAGGAAGCATACGTcATGGCaGGcGTcGGCTCCCCATATGTCTCCCGCCTTCTGGGCATCTGCCTGACATCCACGGTGCAGCTGGTGACACAGCTTATGCCCTATG
Braf-V600E (mouse)	CTTACTGCACCTCAGATATATTTCTTCATGAAGACCTCACGGTAAAAATAGGTGACTTTGGTCTAGCaACgGaGAAgTCgCGGTGGAGTGGGTCCCATCAGTTTGAACAGTTGTCTGGATCTATTTTGTGGATGGTAAGAATTGAGGAGGTATCTCAGTTGGTCAC
Egfr-C799S (mouse)	GATGGCTAGTGTGGACAACCCTCATGTATGCCGCCTCCTGGGCATCTGTCTGACCTCCACTGTCCAGCTCATTACACAGCTCATGCCCTACGGcTcaCTgCTcGACTACGTCCGAGAACACAAGGACAACATTGGCTCCCAGTACCTCCTCAACTGGTGTGTGCAGATTGCAAAGGGCATGAACTACCTGG

Supplementary Table 3. List of primers used for qPCR

Name	Séquence 5’-3’
EGFR_Ctrl_FW	TGCTTCCCCCATTCAGGACT
EGFR_Ctrl_RV	CTCCTTGCACCTCCTCACTG
EGFR_T790M_cbc_FW	CTCTACTGTACAGCTTATAATGCAACTG
EGFR_T790_cbc_RV	CCTTCCCTGATTACCTTTGCGA
EGFR_C797_cbc_FW	CTTCGGGAGTCTGCTTGATTAC
EGFR_C797S_cbc_RV	CCTTATCTCCCCTCCCCGTAT
EGFR_ G724S_cbc_FW	AGGTGACCCTTGTCTCTGTGTT
EGFR_ G724S_cbc_RV	CGCACCGGAACTAAGGACC
EGFR_ G486R_cbc_FW	CGATGGTGACGTCATTATCTCAA
EGFR_ G486R_cbc_RV	ACTAAACAGAAAGCGGTGACT
HER2_ov-A775insYVMA_FW	CCAGCCTTCGACAACCTCTATT
HER2_ov-A775insYVMA _RV	CCAGAGGTTGATTCTCGAGTCAC
MET_ov_FW	AGGCTAGTCCGTTATCAACTTGG
MET_ov_RV	GGTCCATTAGCTGCAAAGATTCC
KRAS_G12D_cbc_FW	TATTAAAAGGTACTGGTGGAGTATTTGAT
KRAS_G12D_cbc_RV	GTCGGCACCAACGACAACT
BRAF_V600E_cbc_FW	ATGCTTGCTCTGATAGGAAAATGAG
BRAF_V600E_cbc_RV	TCCAGCGTGACTTTTCCGTC
EML4-ALK_cbc_FW	CTCGTGGTAACATCAGAACAGAGA
EML4-ALK_cbc_RV	TGCCATGGAGCCTAAGAACTTG
PIK3CA_E545K_cbc_FW	TCTGTCGGAGATAACCAAGCAA

PIK3CA_E545K_cbc_RV	GCTGAGATCAGCCAAATTCAGT
HER2_A775insV-G776C_cbc_FW	CATACGTAATGGCGTTTTGCGTA
HER2_A775insV-G776C_cbc_RV	GGTTTTCCCGGACATGGTCTA
pTripz_5_FW	GACGCTAGCGGATCCATAACT
pTripz_5_RV	GCCTTCGATACCGACACCAT
EGFR-996F	TTGCCGCAAAGTGTGTAACG
EGFR-996R	GTCACCCCTAAATGCCACCG
DUSP6_FW	CTGGAACGAGAATACGGGCG
DUSP6_RV	CTTACTGAAGCCACCTTCCAGG
ETV5_FW	GGACACAGATCTGGCTCACG
ETV5_RV	GGGCATGAAGCACCAGGTTA
SPRY2_FW	GCTCGGAAGTTGGTCTAAAGC
SPRY2_RV	ACATCTGAACTCCGTGATCG
MCL1_FW	TGCTTCGGAAACTGGACATCA
MCL1_FW	TAGCCACAAAGGCACCAAAAG
Braf_V600E_cbc_FW	GGTCTAGCAAGCGAGAAGTGC
Braf_V600_cbc_RV	GCCCTTCAGTGTATTTCTCGTAA
Kras_Ctrl_FW	TAGCTCTCCCGTTCCACAGT
Kras_Ctrl_RV	AGCCGCACTTTACACTGTCC
VIRHD-FW	CAAGGACGACGGCAACTACA
VIRHD-RV	CCTCCTTGAAGTCGATGCCC

Supplementary Table 4. List of the antibodies used

Antibody	Reference	Dilution
Akt (pan) (40D4) - Mouse mAb	#2920, Cell Signaling	1:1,000
c-Myc Clone 9E10, Mouse mAb	11667149001, Roche	1:500
EGF Receptor - Rabbit	#2232, Cell Signaling	1:1,000
eIF4E (C46H6) - Rabbit mAb	#2067, Cell Signaling	1:1,000
FLAG M2, Clone M2 - Mouse mAb	F1804, Sigma	1:500
GAPDH - Mouse mAb	G8795, Sigma	1:5,000
HER2/ErbB2 (D8F12) - Rabbit mAb	#4290, Cell Signaling	1:1,000
Mcl-1 (D35A5) - Rabbit mAb	#5453, Cell Signaling	1:1,000
Met (D1C2) - Rabbit mAb	#8198, Cell Signaling	1:1,000
MNK2 - Rabbit	ab84345, Abcam	1: 1,000
MNK1 - Mouse	sc-133107, Santa Cruz	1:500
p44/42 MAPK (Erk1/2) (L34F12) Mouse mAb	#4696, Cell Signaling	1:1,000
Phospho-p44/42 MAPK (Erk1/2) (Thr202/Tyr204) (D13.14.4E) Rabbit mAb	#4370, Cell Signaling	1:1,000
Phospho-Akt (Ser473) (D9E) - Rabbit mAb	#4060, Cell Signaling	1:1,000
Phospho-EGF Receptor (Tyr1068) (D7A5) - Rabbit mAb	#3777, Cell Signaling	1:1,000
Phospho-eIF4E (Ser209) - Rabbit	#9139, Cell Signaling	1:1,000
Phospho-S6 Ribosomal Protein (Ser240/244)	#2215s, Cell Signaling	1:1,000
Phospho-Stat3 (Tyr705) (D3A7) - Rabbit mAb	#9145, Cell Signaling	1:1,000
Phospho-Stat3 (Tyr705) – Rabbit	#9131, Cell Signaling	1:1,000
Stat3 (124H6) - Mouse mAb	#9139, Cell Signaling	1:1,000
Slug – Rabbit mAb	#9585s, Cell Signaling	1:1,000
Tubulin - Mouse mAb	T5168, Sigma	1:5,000
Vimentin - Rabbit	#3932, Cell Signaling	1:1,000
ß-galactosidase - Goat polyclonal	0856028, MP Biomedicals	1:1,000
GFP - Chicken polyclonal	GFP-1010, Aves Labs	1:2,000
RFP - Rabbit polyclonal	600-401-379, Rockland	1:500
Anti-Goat Alexa Fluor 488	705-545-147, Jackson ImmunoResearch	1:800
Anti-Chicken Cyanine 3	703-165-155, Jackson ImmunoResearch	1:800

Anti-Rabbit Alexa Fluor 647	711-605-152, Jackson ImmunoResearch	1:800
Anti-Chicken Alexa Fluor 647	703-605-155, Jackson ImmunoResearch	1:800
Anti-CD8	#98941, Cell Signaling	1:300
Anti-CD3	MCA1477, Bio-Rad	1:100
Anti-CD68	#97778, Cell Signaling	1:400
Anti-CD86	#19589, Cell Signaling	1:400
