# Supplementary material for: Prolonging lung cancer response to EGFR inhibition by targeting the selective advantage of resistant cells
Source: Nat Commun. 2025 Aug 22;16:7853. doi: 10.1038/s41467-025-61788-w (PMC12373916; doi:10.1038/s41467-025-61788-w)
Supplement: Supplementary file 2 — Description of Additional Supplementary Files [file 41467_2025_61788_MOESM2_ESM.pdf]

Description of Additional Supplementary Files

Title: Supplementary Data 1

Description: Small molecule screen in CRISPR-barcoded PC9 cells treated in combination with gefitinib.

Title: Supplementary Data 2

Description: Gene array analysis of the effects of sorafenib and osimertinib in PC9 cells.

Title: Supplementary Data 3

Description: List of genes used for the the Combo_up and the Combo_down gene signatures.

Title: Supplementary Movie 1:

Description: 3D-imaging of PC9 tumors containing the indicated subpopulations of labelled resistant cells, as described in Fig. 6A. To ensure comparable size for each sample, the tumors from Osimertinib-treated mice were cut in two. Representative of two tumors per condition.

Title: Supplementary Movie 2:

Description: 3D-imaging of YUX-1024 tumors containing a labelled subpopulation of BRAF-V600E resistant cells, as described in Fig. 6C. To ensure comparable size for each sample, the tumors from Osimertinib-treated mice was cut in two. Representative of two tumors per condition.
