# Supplementary material for: Prolonging lung cancer response to EGFR inhibition by targeting the selective advantage of resistant cells
Source: Nat Commun. 2025 Aug 22;16:7853. doi: 10.1038/s41467-025-61788-w (PMC12373916; doi:10.1038/s41467-025-61788-w)
Supplement: Supplementary file 6 — Reporting Summary [file 41467_2025_61788_MOESM6_ESM.pdf]

Corresponding author(s): Luca Grumolato

Last updated by author(s): Jun 2, 2025

Reporting Summary

Nature Portfolio wishes to improve the reproducibility of the work that we publish. This form provides structure for consistency and transparency in reporting. For further information on Nature Portfolio policies, see our Editorial Policies and the Editorial Policy Checklist.

Statistics

For all statistical analyses, confirm that the following items are present in the figure legend, table legend, main text, or Methods section.

n/a	Confirmed	
☐	☒	The exact sample size (n) for each experimental group/condition, given as a discrete number and unit of measurement
☐	☒	A statement on whether measurements were taken from distinct samples or whether the same sample was measured repeatedly
☐	☒	The statistical test(s) used AND whether they are one- or two-sided<br>*Only common tests should be described solely by name; describe more complex techniques in the Methods section.*
☒	☐	A description of all covariates tested
☒	☐	A description of any assumptions or corrections, such as tests of normality and adjustment for multiple comparisons
☐	☒	A full description of the statistical parameters including central tendency (e.g. means) or other basic estimates (e.g. regression coefficient) AND variation (e.g. standard deviation) or associated estimates of uncertainty (e.g. confidence intervals)
☐	☒	For null hypothesis testing, the test statistic (e.g. F, t, r) with confidence intervals, effect sizes, degrees of freedom and P value noted<br>*Give P values as exact values whenever suitable.*
☒	☐	For Bayesian analysis, information on the choice of priors and Markov chain Monte Carlo settings
☒	☐	For hierarchical and complex designs, identification of the appropriate level for tests and full reporting of outcomes
☐	☒	Estimates of effect sizes (e.g. Cohen's d, Pearson's r), indicating how they were calculated

Our web collection on statistics for biologists contains articles on many of the points above.

Software and code

Policy information about availability of computer code

Data collection	ChemiDoc Imaging System (Bio-Rad), QuantStudio Flex PCR System (Thermo Scientific), Beckman Coulter CytoFLEX, Infinite F200 PRO (TECAN), Illumina MiniSeq, Incucyte® S3 Live-Cell Analysis System (Sartorius), Axioscope 7 microscope, Exigo H400 system
Data analysis	Prism 4 software from GraphPad (GraphPad Software Inc), Microsoft Excel LTSC MSO (16.0.14332.21031) 64 bits, Image Lab Software (version 6.0.1; Bio-Rad), QuantStudio Design and Analysis Software (v1.4.3; Thermo Scientific), CytExpert software (v2.5.0.77; Beckman Coulter), Imaris software (v10.0.2; Bitplane), Incucyte software (v2021C; Sartorius), FlowJo software (V10.10.0, Becton Dickinson).

For manuscripts utilizing custom algorithms or software that are central to the research but not yet described in published literature, software must be made available to editors and reviewers. We strongly encourage code deposition in a community repository (e.g. GitHub). See the Nature Portfolio guidelines for submitting code & software for further information.

Data

Policy information about availability of data

All manuscripts must include a data availability statement. This statement should provide the following information, where applicable:

- Accession codes, unique identifiers, or web links for publicly available datasets
- A description of any restrictions on data availability
- For clinical datasets or third party data, please ensure that the statement adheres to our policy

Gene expression data from renal cell carcinoma patients30 were retrieved from Gene Expression Omnibus (GEO) GSE180925 [https://www.ncbi.nlm.nih.gov/geo/query/acc.cgi?acc=GSE180925]. RNA-seq data from thyroid cancer patients31 were retrieved from BioProject (Submission ID: SUB6216503; BioProject ID:PRJNA563018) [http://www.ncbi.nlm.nih.gov/bioproject/563018]. The microarray datasets generated in this study were deposited into GEO (GSE179192) [https://www.ncbi.xyz/geo/query/acc.cgi?acc=GSE179192]. The remaining data are available within the Article, Supplementary Information or Source Data file. Source data are provided with this paper.

Research involving human participants, their data, or biological material

Policy information about studies with human participants or human data. See also policy information about sex, gender (identity/presentation), and sexual orientation and race, ethnicity and racism.

Reporting on sex and gender	*Use the terms sex (biological attribute) and gender (shaped by social and cultural circumstances) carefully in order to avoid confusing both terms. Indicate if findings apply to only one sex or gender; describe whether sex and gender were considered in study design; whether sex and/or gender was determined based on self-reporting or assigned and methods used. Provide in the source data disaggregated sex and gender data, where this information has been collected, and if consent has been obtained for sharing of individual-level data; provide overall numbers in this Reporting Summary. Please state if this information has not been collected. Report sex- and gender-based analyses where performed, justify reasons for lack of sex- and gender-based analysis.*
Reporting on race, ethnicity, or other socially relevant groupings	*Please specify the socially constructed or socially relevant categorization variable(s) used in your manuscript and explain why they were used. Please note that such variables should not be used as proxies for other socially constructed/relevant variables (for example, race or ethnicity should not be used as a proxy for socioeconomic status). Provide clear definitions of the relevant terms used, how they were provided (by the participants/respondents, the researchers, or third parties), and the method(s) used to classify people into the different categories (e.g. self-report, census or administrative data, social media data, etc.) Please provide details about how you controlled for confounding variables in your analyses.*
Population characteristics	*Describe the covariate-relevant population characteristics of the human research participants (e.g. age, genotypic information, past and current diagnosis and treatment categories). If you filled out the behavioural & social sciences study design questions and have nothing to add here, write "See above."*
Recruitment	*Describe how participants were recruited. Outline any potential self-selection bias or other biases that may be present and how these are likely to impact results.*
Ethics oversight	*Identify the organization(s) that approved the study protocol.*

Note that full information on the approval of the study protocol must also be provided in the manuscript.

Field-specific reporting

Please select the one below that is the best fit for your research. If you are not sure, read the appropriate sections before making your selection.

[x] Life sciences [ ] Behavioural & social sciences [ ] Ecological, evolutionary & environmental sciences

For a reference copy of the document with all sections, see nature.com/documents/nr-reporting-summary-flat.pdf

Life sciences study design

All studies must disclose on these points even when the disclosure is negative.

Sample size	Statistical tests were not performed to choose sample size.<br>For in vitro experiments, at least 3 biological repeats were conducted to ensure the reproducibility of our results.<br>For in vivo experiments, 5-10 mice per group for PC9, BEM4 and YUX-1024 derived xenografts and 5-16 mice per group for BEM-5 tumors; 2 mice per group for iDISCO experiments were used to ensure the reproducibility of our results.<br>For qPCR analysis, 4-5 replicates per conditions were used, data are expressed as mean ± SEM.
Data exclusions	In the Kaplan-Meier plot in Fig. 6E, 1 Ctrl and 2 Osim mice were censored because they were found dead in their cage or they lost > 10% weight).
Replication	All the in vitro experiments (except for gene array and highly complex DNA barcodes) were independently repeated at least three times, with comparable results obtained each time. The results of all replicate experiments have been provided as an excel file in the Supplementary

	Information.
Randomization	For in vivo studies, once the tumors reached a given size (indicated in the text for each experiment), the mice were randomly assigned to treatment with vehicle, sorafenib, osimertinib or the combination. For other experiments, no randomization was performed.
Blinding	The investigators were not blinded to allocation during the experiments and the analysis due to feasibility. The study does not involve clinical trials or histopathology analysis of clinical samples and blinding was not required.

Reporting for specific materials, systems and methods

We require information from authors about some types of materials, experimental systems and methods used in many studies. Here, indicate whether each material, system or method listed is relevant to your study. If you are not sure if a list item applies to your research, read the appropriate section before selecting a response.

Materials & experimental systems

n/a	Involved in the study
☐	☒ Antibodies
☐	☒ Eukaryotic cell lines
☒	☐ Palaeontology and archaeology
☐	☒ Animals and other organisms
☒	☐ Clinical data
☒	☐ Dual use research of concern
☒	☐ Plants

Methods

n/a	Involved in the study
☒	☐ ChIP-seq
☐	☒ Flow cytometry
☒	☐ MRI-based neuroimaging

Antibodies

Antibodies used

Antibodies used for immunoblotting :

Akt (pan) (40D4) - Mouse mAb #2920, Cell Signalling 1:1,000
c-Myc (9E10), Mouse mAb 11667149001, Roche 1:500
EGF Receptor - Rabbit #2232, Cell Signalling 1:1,000
eIF4E (C46H6) - Rabbit mAb #2067, Cell Signalling 1:1,000
FLAG M2, Clone M2 - Mouse mAb F1804, Sigma 1:500
GAPDH - Mouse mAb G8795, Sigma 1:5,000
HER2/ErbB2 (D8F12) - Rabbit mAb #4290, Cell Signalling 1:1,000
Mcl-1 (D35A5) - Rabbit mAb #5453, Cell Signalling 1:1,000
Met (D1C2) - Rabbit mAb #8198, Cell Signalling 1:1,000
MNK2 - Rabbit ab84345, Abcam 1: 1,000
MNK1 - Mouse sc-133107, Santa Cruz 1:500
p44/42 MAPK (Erk1/2) (L34F12) Mouse mAb #4696, Cell Signalling 1:1,000
Phospho-p44/42 MAPK (Erk1/2) (Thr202/Tyr204)
(D13.14.4E) Rabbit mAb
#4370, Cell Signalling 1:1,000
Phospho-Akt (Ser473) (D9E) - Rabbit mAb #4060, Cell Signalling 1:1,000
Phospho-EGF Receptor (Tyr1068) (D7A5) - Rabbit mAb #3777, Cell Signalling 1:1,000
Phospho-eIF4E (Ser209) - Rabbit #9139, Cell Signalling 1:1,000
Phospho-S6 Ribosomal Protein (Ser240/244) #2215s, Cell Signalling 1:1,000
Phospho-Stat3 (Tyr705) (D3A7) - Rabbit mAb #9145, Cell Signalling 1:1,000
Phospho-Stat3 (Tyr705) – Rabbit #9131, Cell Signalling 1:1,000
Stat3 (124H6) - Mouse mAb #9139, Cell Signalling 1:1,000
Slug – Rabbit mAb #9585s, Cell Signalling 1:1,000
Tubulin - Mouse mAb T5168, Sigma 1:5,000
Vimentin - Rabbit #3932, Cell Signalling 1:1,000``

Antibodies used for iDISCO:

ß-galactosidase - Goat polyclonal 0856028, MP Biomedicals 1:1,000
GFP - Chicken polyclonal GFP-1010, Aves Labs 1:2,000
RFP - Rabbit polyclonal 600-401-379, Rockland 1:500
Anti-Goat Alexa Fluor 488 705-545-147, Jackson ImmunoResearch 1:800
Anti-Chicken Cyanine 3 703-165-155, Jackson ImmunoResearch 1:800
Anti-Rabbit Alexa Fluor 647 711-605-152, Jackson ImmunoResearch 1:800
Anti-Chicken Alexa Fluor 647 703-605-155, Jackson ImmunoResearch 1:800

Antibodies used for IHC:

	Anti-CD8 #98941, Cell Signaling 1:300<br>Anti-CD3 MCA1477, Bio-Rad 1:100<br>Anti-CD68 #97778, Cell Signaling 1:400<br>Anti-CD86 #19589, Cell Signaling 1:400
Validation	All primary and secondary antibodies used in this study are commercially available and have been validated for used applications in human cells by the manufacturers. Appropriate positive and negative controls were included in our experiments.

Eukaryotic cell lines

Policy information about cell lines and Sex and Gender in Research

Cell line source(s)	Human embryonic kidney 293T cells were obtained from ATCC (CRL-3216) ; PC9 cells (NSCLC, EGFR-Ex19Del) were obtained from ECACC (distributed by Sigma-Aldrich, 90071810); HCC4006 (NSCLC, EGFR-Ex19Del, CRL-2871) and H1975 cells (NSCLC, EGFR-L858R/T790M, CRL-5908) were obtained from ATCC; H3122 cells (NSCLC, EML4-ALK, 300484) were obtained from Cytion; HCC827 cells (NSCLC, EGFR-Ex19Del) and H358 (NSCLC, KRAS-G12C) were a gift from Pr. J. Minna, UT Southwestern Medical Center, Dallas (USA); HCC827-GR6 were a gift from Pr. P. Jänne, Dana-Farber Cancer Institute, Boston (USA); HCA-46 cells (CRC, 07031601) were obtained from ECACC; CCK81 cells (CRC) were obtained from HSRRB (Japan); LIM1215 cells (Whitehead et al., 1985) were obtained from Prof. R. Whitehead, Vanderbilt University, Nashville (USA), with permission from the Ludwig Institute for Cancer Research, Zurich (Switzerland). NSCLC PDC lines YU-1150 (EGFR-L858R/T790M) and YUX-1024 (EGFR-L858R) were described previously (Yun et al., 2019). BALB-3T3 cells were derived from BALB/c embryos (Aaronson and Todaro, 1968).
Authentication	All the cell lines (except 293T, H3122, YU-1150, YUX-1024 and BALB-3T3) were authenticated by STR testing (Eurofins).
Mycoplasma contamination	Cells were examined for potential mycoplasma contamination routinely and all cell cultures were negative of mycoplasma contamination before experiments.
Commonly misidentified lines<br>(See ICLAC register)	No commonly misidentified lines were used in this study.

Animals and other research organisms

Policy information about studies involving animals; ARRIVE guidelines recommended for reporting animal research, and Sex and Gender in Research

Laboratory animals	6-8 week old SCID (CB-17/Icr-Prkdc scid/scid/Rj) and BALB/c (BALB/cJRj) mice were purchased from Janvier labs. All mice were housed in pathogen-free facilities (SOPF). All mice were fed with filtered water and sterilized food under standard conditions (temperature 22°C +/- 0,5 ; relative hygrometry : 55%)
Wild animals	Wild animal were not involved in the study.
Reporting on sex	For this study, we used both male and female mice.
Field-collected samples	No field-collected samples were used in this study.
Ethics oversight	Animal experiments were approved by Mount Sinai's Animal Care and Use Committee (IACUC) or the French Regional Ethics Committees and the Ministry of Education, Research and Innovation (project n°19253-201903191709966 v1 and authorization n° 11752 – 16/10/18), and they were performed in accordance with the European Committee Council Directive (2010-63-EU).

Note that full information on the approval of the study protocol must also be provided in the manuscript.

Flow Cytometry

Plots

Confirm that:

- [x] The axis labels state the marker and fluorochrome used (e.g. CD4-FITC).
- [x] The axis scales are clearly visible. Include numbers along axes only for bottom left plot of group (a 'group' is an analysis of identical markers).
- [ ] All plots are contour plots with outliers or pseudocolor plots.
- [x] A numerical value for number of cells or percentage (with statistics) is provided.

Methodology

Sample preparation	Cell cycle was analysed by flow cytometry analysis using the Click-iT EdU cell proliferation assay. Cells were labelled with EdU for 1h, followed by fixation, permeabilization and staining with DAPI. To measure apoptosis, after trypsinization, the cells were centrifuged at 1000 rpm for 5 min and washed twice in pre-cooled PBS. The cells were re-suspended in 1X binding

	buffer and mixed with Annexin V-FITC and PI by incubation in the dark at room temperature for 15 min. Parental or Flag-EGFR_Ex19Del-T2A-GFP (VIRSP)-transduced PC9 cells, treated with or without sorafenib (5 µM) for three days, were trypsinized, washed and fixed with 4% paraformaldehyde.
Instrument	CytoFLEX Flow Cytometer
Software	CytExpert software (Beckman Coulter)
Cell population abundance	No cell sorting was performed in this manuscript.
Gating strategy	Live cells were separated from debris and dead cells with a side scatter area (SSC-A) (y) versus forward scatter area (FSC-A) (x) dot plot. Then, a dot plot forward scatter height (FSC-H) (y) versus FSC-A (x) was used to discriminate doublets, and finally 20,000 singlet cells were analysed for GFP levels in an histogram illustrating the cell count (y) versus fluorescein isothiocyanate/GFP fluorescence levels (FITC-A) (x).

☐ Tick this box to confirm that a figure exemplifying the gating strategy is provided in the Supplementary Information.
